# Sex-specific differences in cardiac function, inflammation and injury during early polymicrobial sepsis

**DOI:** 10.1186/s40635-022-00454-7

**Published:** 2022-06-20

**Authors:** Sophie L. M. Walker, Chand Muthoo, Jenifer Sanchez, Ana Gutierrez Del Arroyo, Gareth L. Ackland

**Affiliations:** Translational Medicine and Therapeutics, William Harvey Research Institute, Queen Mary University of London, London, EC1M 6BQ UK

**Keywords:** Sepsis, Gender, Leukocyte, Cardiac dysfunction

## Abstract

**Background:**

Sex differences in sepsis are underexplored and incompletely understood. Cardiac function in early sepsis is pivotal in determining survival; hyperdynamic left ventricular ejection fraction is associated with higher mortality. Female sex may be cardioprotective, but variable experimental findings have not controlled for hypovolaemia. Sex-specific local cardiac versus peripheral inflammation in causing cardiovascular dysfunction also remain unclear. We therefore examined whether there are sex-specific differences in cardiac function in early sepsis, controlling for volaemic status and sex-specific differences in the peripheral inflammatory response initiated by tumour necrosis factor (TNFα).

**Methods:**

We used an experimental polymicrobial sepsis (faecal slurry) model titrated to minimise hypovolaemia as a confounding factor. We quantified cardiac function (transthoracic cardiac echocardiography) 1 week before, and 18 h after, sepsis. Cardiac injury (troponin I), inflammation and immune cell infiltration (flow cytometry) were quantified in naïve and septic female and male mice 18 h after sepsis. To evaluate the sex-specific influence of TNFα derived from peripheral leukocytes, we repeated the experiments in iRHOM2^−/−^ mice that are unable to shed TNFα exclusively from circulating leucocytes.

**Results:**

Serum troponin I increased to 1.39 ± 0.38 ng mL^−1^ (from undetectable levels in controls) 18 h after onset of normovolaemic sepsis to a similar extent in both sexes. Stroke volume in male mice increased by 8 µL [(3–13); *p* = 0.004], compared to individualised pre-sepsis values. By contrast, stroke volume remained at baseline levels in females [mean difference: 4 µL (− 1 to 9)]. Messenger RNA levels of markers for cardiac injury/inflammation after sepsis (real-time polymerase-chain reaction) were elevated in male wild-type mice compared to female wild types (*n* = 10/sex), with higher cardiac mRNA levels of atrial natriuretic peptide, inflammation (TNFα) and oxidative stress (superoxide dismutase-1), although serum troponin I values were similarly elevated. Flow cytometry analysis of cardiac tissue showed doubling of CD4 + leukocyte infiltration in male mice. Sex-specific cardiac physiologic differences were similar in iRHOM2^−/−^ mice that are unable to shed TNFα exclusively from leucocytes.

**Conclusions:**

In early normovolaemic polymicrobial sepsis, a relative hyperdynamic response develops in male mice. Myocardial stress/injury after early sepsis is limited in females, with less cardiac infiltration of CD4 + leukocytes but independent of shedding of TNFα from peripheral circulating leukocytes.

**Supplementary Information:**

The online version contains supplementary material available at 10.1186/s40635-022-00454-7.

## Take-home message

- Sex differences in sepsis are underexplored, even though female sex appears to be cardioprotective in early sepsis.
- In early normovolaemic polymicrobial sepsis, hyperdynamic response occurs in males, whereas myocardial stress/injury is limited in females, in part mediated by less cardiac infiltration of CD4 + leukocytes and independent of shedding of TNFα from peripheral circulating leukocytes.

**Supplementary Information:**

The online version contains supplementary material available at 10.1186/s40635-022-00454-7.

## Background

The severity and prognosis of sepsis may be more favourable for females [1]. The mechanisms underlying these sex differences are incompletely understood, suggesting that an enhanced understanding of sex-dependent biologic responses are likely to improve translational research success [2]. Preservation of normal cardiac function and/or absence of myocardial injury (as evidenced by elevated levels of circulating troponin) are independently associated with reduced mortality in early sepsis and appears to correlate with the lower number of females requiring critical care and/or dying [3, 4]. By contrast, hyperdynamic left ventricular ejection fraction is associated with higher mortality [5–7]. Although selection bias may account for this apparent sex-related discrepancy in critical care utilisation [1], several biologic reasons seem more likely to underpin such sex-specific epidemiologic outcomes in sepsis [2].

Laboratory studies in sepsis show that males appear less able to respond positively to rapid fluid resuscitation in early sepsis [8]. Male cardiac dysfunction may be attributable to sex-related differences in beta-adrenoreceptor physiology [4, 9] and/or oestrogen-related immunomodulation [2], which may reduce pivotal pro-inflammatory cytokines including TNFα that directly injure the heart [10]. Early cardiac dysfunction in experimental sepsis can be prevented by anti-TNFα therapy [11].

In sepsis, the source of TNFα remains unclear, since cardiomyocytes can directly generate inflammatory mediators [12–14]. Depending on the relative expression of the injurious TNF receptor 1 (TNFR1) versus the cardioprotective TNF receptor 2 (TNFR2) in cardiomyocytes, TNFα may ameliorate, or accelerate, cardiac failure [15]. Shedding of TNFα and CD62L from the cell surface by ADAM17 (ADAM Metallopeptidase Domain 17) is required to initiate and sustain systemic inflammation. Rhomboid serine proteases (iRhoms) control the trafficking and maturation of ADAM17 [16, 17]. iRHOM2 deficiency in circulating immune cells prevents shedding of TNFα, resulting in markedly lower serum TNFα after a systemic lipopolysaccharide challenge [18].

Here, we have re-examined the role of sex in early cardiac injury and function following sepsis. We developed a murine model of isovolaemic sepsis to compare physiologic and inflammatory cardiac features between sexes to establish the respective roles of cardiac versus peripheral inflammation. This study tested the hypothesis that lack of TNF shedding from peripheral leukocytes would mitigate sex-specific differences in the cardiac response to early normovolaemic sepsis.

## Methods

All procedures complied with the UK Home Office Legislation (1986 Animal Procedures Act) and were approved by the Institutional Animal Care and Use Committee at William Harvey Research Institute. Experiments were performed in accord with ARRIVE guidelines (Additional file 1: Table S1). Female and male wild-type (C57Bl/6 background) and iRhom2^−/−^ littermates aged 8–12 weeks were used, as detailed previously [19], under 12 h dark/light cycles and provided with enrichment, water and food ad libitum.

### Murine experimental sepsis

Polymicrobial sepsis was induced in conscious mice that were not starved before a single intraperitoneal injection of cecal slurry (4 μL/g body weight, amounting to 1 mL sterile saline) collected from three donor wild-type C57/Bl6 mice [20]. No analgesia was administered with the intraperitoneal injection. Mice had free access to drinking water during the experiment. The cecal slurry dose was calibrated in preliminary experiments to avoid hypovolaemia and ensure survival for at least 72 h [20], in accord with pre-clinical sepsis guidelines [21]. Grading of clinical appearance after sepsis was recorded using a validated scale which correlates closely with the degree of organ failure (Additional file 1: Supplementary data 2). Naive mice were littermate controls that received no intervention.

### Experimental protocol

**Fig. 1:**
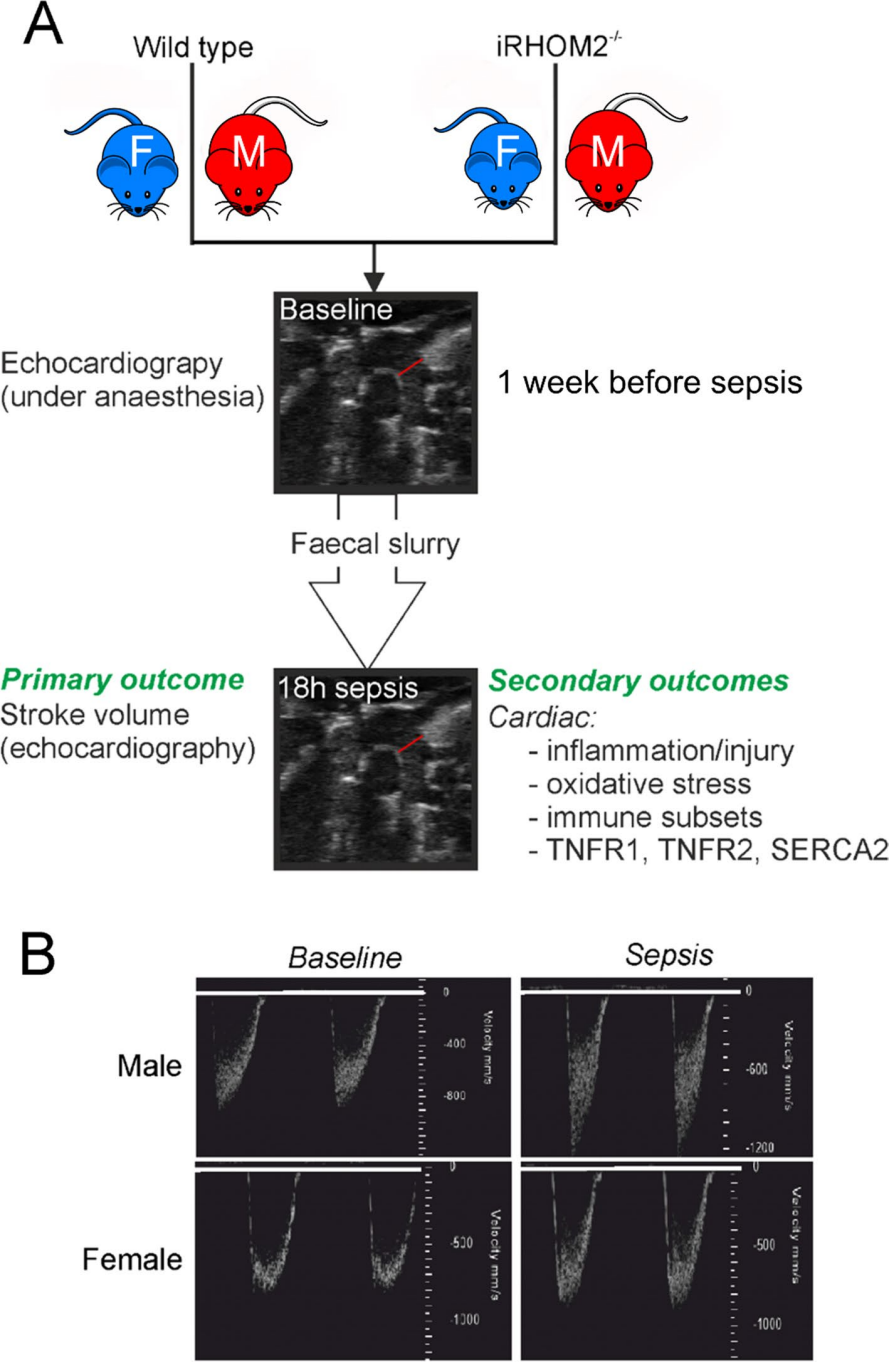
Experimental protocol. **A** Summary of protocol, including primary and secondary outcomes. Colours for female/male mice for each genotype are used throughout the results section for clarity. **B** Typical echocardiography results in one female and one male mouse, before and 18 h after onset of sepsis showing pulmonary velocity time interval echocardiographic measurement

Mice underwent cardiac echocardiography 2 weeks before the induction of polymicrobial sepsis, which was induced at the same time of day to minimise circadian influences (Fig. 1). Cardiac echocardiography assessment was repeated 18 h after the onset of sepsis under isoflurane anaesthesia, after which point mice were euthanised by cervical dislocation.

### Cardiac injury—serum troponin I

Cardiac TnI concentration in 100 µL serum samples was measured using a mouse-specific enzyme-linked immunoassay (Life Diagnostics, Inc., West Chester, PA). Cardiac troponin I, purified from mouse hearts was used to construct a standard curve by plotting absorbance values of the standards versus log10 of the concentration by colorimetric microplate reader (Dynex technology, West Sussex, UK). The standard curve was fitted to a four-parameter logistic regression equation (*x* axis = log10 concentration) to determine the concentration TnI in serum samples.

### Cardiac function

Cardiac function was assessed by transthoracic echocardiography under 2% isoflurane anaesthesia with temperature maintained at 37 °C (Vevo 3100 transthoracic echocardiography, FujiFilm VisualSonics, Toronto, Canada). Imaging was completed within 15 min. At least 10 cardiac cycles were collected before averaging cardiac parameters. All cardiac imaging data were assessed by an assessor masked to the experimental details. Ejection fraction, fractional shortening, left ventricular end-diastolic volume, stroke volume, and cardiac output were measured from M‐mode measurements. Both pulsed wave Doppler of the pulmonary arterial blood flow and aortic flow were undertaken.

### Cardiac immunophenotyping

Flow cytometry was used to compare between each genotype and sex (antibodies and gating strategy are detailed in Additional file 1: Supplementary data 3, 4). Whole hearts were removed and washed in PBS. Finely minced tissue was then digested for 30 min at 37 °C [Krebs–Henseleit solution containing collagenase (*Clostridium histolyticum* Type IA; C9891, Sigma-Aldrich), Type XI collagenase (C7657, Sigma-Aldrich), bovine pancreatic deoxyribonuclease I (D4263, Sigma-Aldrich) and hyaluronidase Type IV from bovine testes (H3884, Sigma-Aldrich)]. Digestates were then passed through a 70-micron cell strainer and reconstituted with PBS prior to density gradient centrifugation (Ficoll-Paque PLUS (17-1440-02, GE Healthcare Life Sciences). Isolated immune cells were washed again with phosphate-buffered saline, incubated with an antibody cocktail comprising CD45-FITC (clone REA737), CD8a-PerCPVio700 (clone 53-6.7), CD4-APC (clone GK1.5), Ly6G-APCVio770 (clone REA526), Ly6C-VioBlue (clone REA796), CD45B220-VioGreen (clone REA755), CD62L-Brilliant Violet 785 (clone MEL-14), F4/80-PE (clone REA126), CD11b-PEVio615 (clone REA592) and CXCR2-PEVio770 (clone REA942). Cells were fixed with 100 μL 2% paraformaldehyde.

### Cardiac contractility

RNA was extracted to quantify mRNA levels in cardiac ventricular tissue using murine specific, validated primers (Additional file 1: Supplementary data 5). We quantified messenger RNA expression of sarco/endoplasmic reticulum Ca(2+)-ATPase (SERCA2), which regulates cardiomyocyte SR Ca2 + haemostasis) and hence is used widely as a proxy for cardiac contractility [22]. SERCA2 is required to maintain cardiac contractility during experimental sepsis and is associated with sex-specific differences [3, 23]. We also quantified messenger RNA levels of NOX-2, which mediates cardiomyocyte-specific enhancement of contractility during acute stress [24]. The comparative Ct method (2-∆∆Ct) was used, with hypoxanthine-guanine phosphoribosyltransferase (HPRT) or cyclophilin as the housekeeping gene. Key regulatory proteins that determine contractility were quantified by immunoblots from cell lysates prepared from cardiac ventricular tissue in mice 18 h after the onset of polymicrobial sepsis [25]. Phosphorylation of troponin I (phospho-Troponin I (Ser23/24); Cell Signaling Technology, #4004), phospholamban (rabbit monoclonal, Cell Signaling Technology #14562) and SERCA2 (rabbit monoclonal, Cell Signaling Technology, #9580) were quantified. Proteins were resolved on SDS-PAGE gels. Densitometry determinations were calculated as the ratio between the protein and β-actin protein expression.

### TNFα quantification

TNFα was measured in duplicate peritoneal lavage samples using a murine specific TNFα Quantikine solid-phase immunoassay enzyme-linked immunoassay (MTA00B, R&D systems, Bio-techne, Abingdon, UK; intra-assay CV% = 3.1%; inter-assay CV% = 7%). TNFα concentration was calculated using the standard curve following quantification by colorimetric microplate reader (Dynex technology, West Sussex, UK). Semi-quantitative analysis by immunoblotting for cardiac TNFα was also conducted (#01164, Millipore, Leuven Belgium).

### Cardiac inflammation

We quantified mRNA levels in cardiac ventricular tissue using murine specific primers (Additional file 1: Supplementary data 5) for atrial natriuretic peptide (a robust cardiac biomarker during short-term cardiac stress), [26] Nuclear factor-ĸ B (NF-ĸB) [27], and superoxide dismutase (SOD-1), cytosolic deficiency of which renders cardiac tissue vulnerable to greater injury [28]. Since cardiomyocyte TNFR1 deficiency is protective, whereas cardiomyocyte TNFR2 deficiency is deleterious [15], we assessed the left ventricular TNFR1:R2 ratio which reflects the balance between TNFR-mediated pro-survival versus death signalling pathways [15].

### Statistical analyses

Mean (standard deviation), median (25–75th centile) or *n* (%) values are presented. Data were analysed using GraphPad Prism version 8.0.0 (GraphPad Software, Inc., La Jolla, CA, USA). Sidak’s multiple comparison post hoc test was used for one or two-way ANOVA analysis, as indicated in text. *p* values ≤ 0.05 were taken as statistically significant.

### Sample size estimations

Sample size calculations were derived using PASS (NCSS, Kaysville, Utah, USA). On the basis that transthoracic echocardiography 18 h after the onset of sepsis would demonstrate a change ≥ 10% in stroke volume compared to individualised (non-septic) baseline values, ≥5 mice of each sex would be required (α = 0.01; 1-β = 0.1), with each mouse serving as its own control. Similar changes in ejection fraction have been reported after [hypovolaemic] caecal ligation and puncture [29]. For measures of inflammation, based on a fourfold reduction in mean serum TNF in iRHOM2 knockout mice after challenge with lipopolysaccharide [18], at least three mice/genotype were required to have a 90% chance of detecting (*p* < 0.05), a difference in inflammatory measures (based on mean TNF 1200 ± 300 pg mL^−1^ in wild-type mice, compared with 300 ± 100 pg mL^−1^ in iRHOM2 mice in original iRHOM2 manuscript [18]).

## Results

### Clinical phenotype after experimental polymicrobial sepsis

**Fig. 2:**
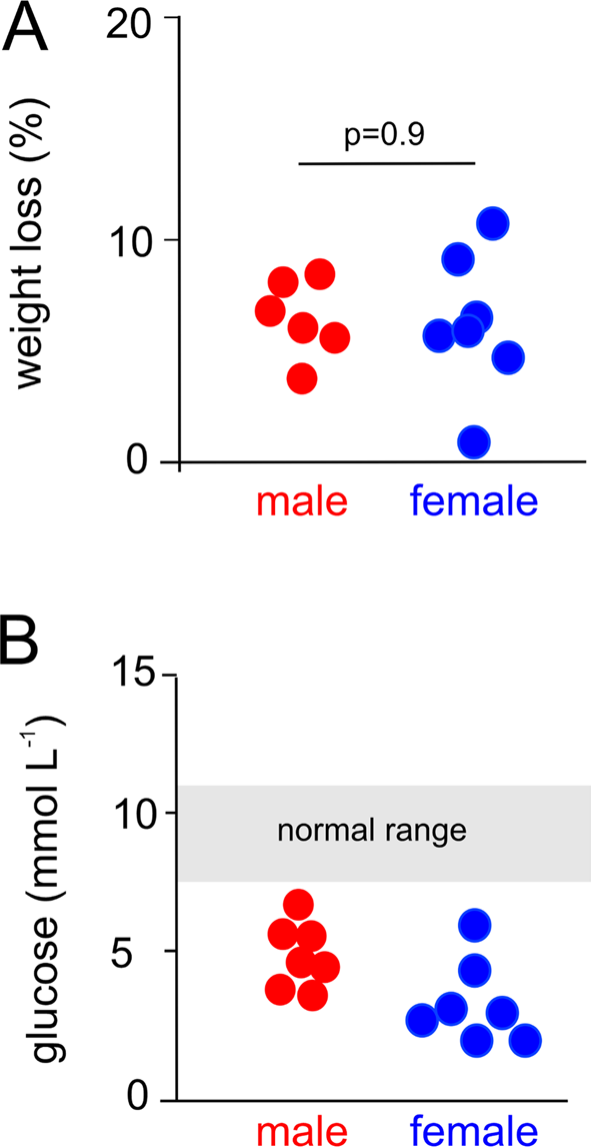
Weight loss and blood glucose levels in polymicrobial sepsis. **A** % weight loss compares weight at 18 h after onset of sepsis versus pre-sepsis weight for individual mice of each sex (*n* = 6). **B** Venous blood glucose (Roche Accuchek monitor) compared at 18 h after onset of sepsis (*n* = 6/sex) versus indicates normal range for blood glucose in naïve male and female mice as indicated by grey area (*n* = 12)

Polymicrobial sepsis resulted in similar symptoms/signs of mild–moderate severity in both sexes, with organ injury in the form of myocardial damage evident from elevation of troponin levels that were similar between male and female mice (Table 1). Weight loss and lower blood glucose levels (*p* < 0.001) were similar between male (mean glucose: 5.4 ± 1.2 mmol/L) and female (mean glucose: 3.7 ± 1.4 mmol/L) mice 18 h after the onset of polymicrobial sepsis (Fig. 2). No mice died within 18 h of sepsis. Serum troponin I was below the limits of detection in naïve mice, but elevated 18 h after sepsis to similar levels in male and female mice (Table 1).

**Table 1:** Clinical severity and cardiac injury following isovolaemic polymicrobial murine sepsis

	Wild type		*p* value	iRHOM2 knockout		*p* value
	Male	Female		Male	Female	
Clinical severity						
Unaffected	0	0		0	0	
Mild	4	4	0.99	4	4	0.99
Moderate	2	2		2	2	
Severe	0	0		0	0	
Cardiac injury						
Troponin (ng mL^−1^)	1.49 ± 0.20	1.46 ± 0.11	0.34	1.24 ± 0.46	1.40 ± 0.15	0.36

Troponin values are mean ± SD

### Sex-specific cardiac physiology in early experimental murine sepsis

**Fig. 3:**
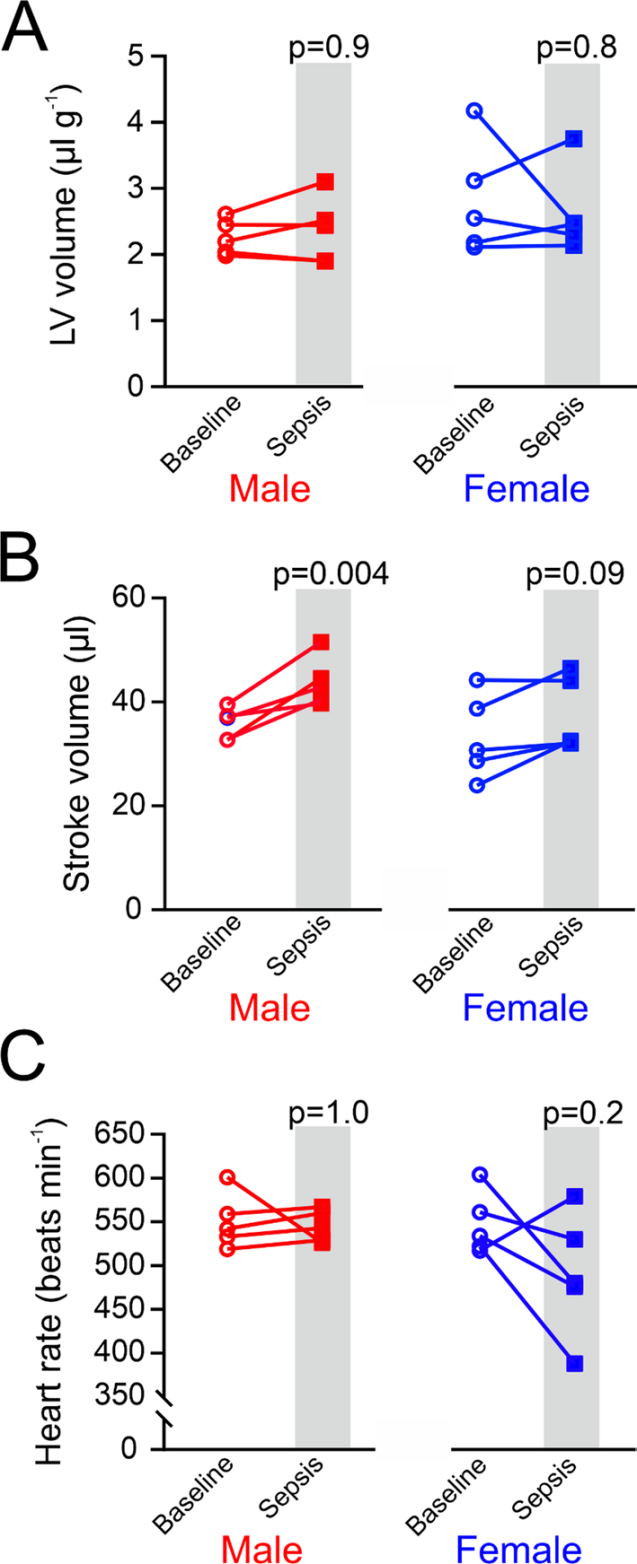
Serial echocardiography in wild-type female and male mice before and after polymicrobial sepsis. **A** Left ventricular volume (standardised by body weight) in wild-type male and female mice (*n* = 5/sex), as indicator of preload 18 h after the onset of sepsis. **B** Stroke volume in male and female mice (*n* = 5/sex). Stroke volume increased only in both wild-type male mice 18 h after the onset of sepsis. **C** Heart rate in wild-type male and female mice, as indicator of preload 18 h after the onset of sepsis. **D** Data for each individual mouse were compared to its own pre-sepsis baseline reading, measured ~ 1 week before the onset of sepsis. Individual *p* values on each panel refer to post hoc testing after ANOVA testing for sex *x* time interaction

Our model of polymicrobial sepsis did not alter volaemic status, with similar end-diastolic left ventricular volumes in males (2.3 ± 0.3 µL g^−1^) and female (2.8 ± 0.9 µL g^−1^) a.u.) before and 18 h after sepsis (male: 3.4 ± 0.5 µL g^−1^ versus female: 2.6 ± 0.7 µL g^−1^; Fig. 3A). Stroke volume increased only in male wild-type mice after polymicrobial sepsis (from 36 ± 3 µL to 44 ± 5 µL; Fig. 3B), with end-diastolic volume and heart rate remaining at baseline levels (Fig. 3C; Additional file 1). Stroke volume after sepsis was similar between sexes (*p* = 0.22).

### Sex-specific cardiac injury and inflammation after experimental polymicrobial sepsis in wild-type mice

**Fig. 4:**
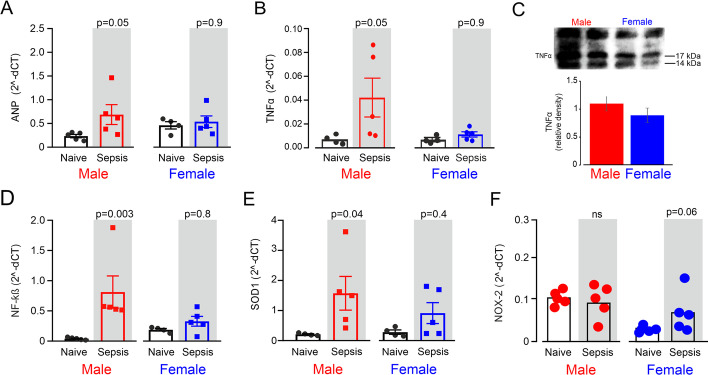
Messenger RNA signatures of cardiac injury/inflammation after polymicrobial sepsis. **A** mRNA expression in left ventricle of atrial natriuretic peptide (ANP). Wild-type male mice (*n* = 5) showed higher ANP gene transcription relative to naive sex and age-matched naïve males 18 h after the onset of sepsis and compared to age-matched females (*n* = 5). **B** Cardiac inflammation as indicated by mRNA expression of TNFα in left ventricle. Only male wild-type mice (*n* = 5) showed higher TNFα gene transcription 18 h after the onset of sepsis, compared to age-matched females (*n* = 5). **C** Semi-quantitative analysis by immunoblotting for cardiac TNFα in four mice/group. **D** Cardiac inflammation as indicated by mRNA expression of NF-kappa B in left ventricle. Only male wild-type mice (*n* = 5) showed higher NF-kappa B gene transcription 18 h after the onset of sepsis, compared to age-matched females (*n* = 5). **E** Cardiac oxidative stress as indicated by mRNA expression of Superoxide dismutase-1 in left ventricle. Only male wild-type mice (*n* = 5) showed higher SOD-1 gene transcription 18 h after the onset of sepsis, compared to age-matched females (*n* = 5). **F** mRNA expression of nicotinamide adenine dinucleotide phosphate oxidase 2 (NADPH oxidase 2 or Nox2) in left ventricle. (*n* = 5/group). Individual *p* values on each panel refer to post hoc testing after ANOVA testing for genotype *x* sepsis interaction

Cardiac ANP mRNA increased from 0.24 ± 0.08 to 0.69 ± 0.47 a.u. in male mice (*p* = 0.05) after sepsis, but remained unchanged in female mice (0.46 ± 0.15 to 0.54 ± 0.27 a.u.; *p* = 0.90). Cardiac ANP mRNA was similar between sexes 18 h after sepsis (*p* = 0.68; Fig. 4A). This was accompanied by higher mRNA expression of left ventricular TNFα (Fig. 4B) in male wild-type mice (0.04 ± 0.04, compared to female 0.01 ± 0.01 a.u.; *p* = 0.05). Semi-quantitation of cardiac protein levels of left ventricular TNFα by immunoblot were also higher in males (Fig. 4C). Expression levels of left ventricular NF-ĸB mRNA (male: 0.81 ± 0.6 a.u. versus female: 0.34 ± 0.18 a.u.) and SOD-1 mRNA (male: 1.5 ± 1.2 a.u. versus female: 0.90 ± 0.76 a.u.) and were also higher in males (Fig. 4D, E). Sepsis resulted in higher NOX-2 mRNA levels only in female mice (Fig. 4F).

### Sex-specific cardiac infiltration by leukocytes after experimental polymicrobial sepsis in wild-type mice

**Fig. 5:**
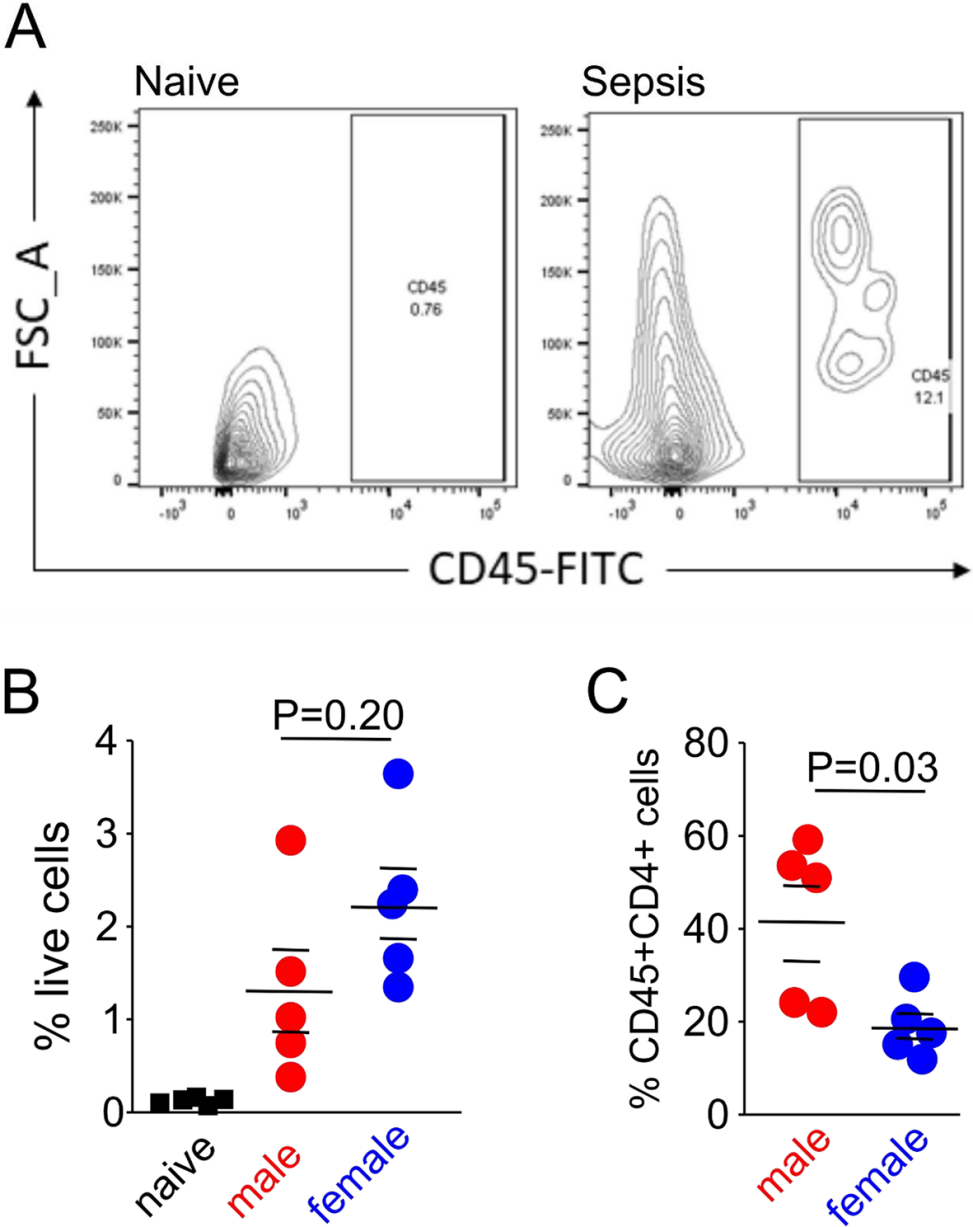
Cardiac immune infiltrates after polymicrobial sepsis. **A** Dot plot showing CD45^+^ immune cells in naïve and septic heart tissue after digestion/density centrifugation. **B** Proportion of CD45^+^ immune cells in hearts obtained from wild-type mice after 18 h of polymicrobial sepsis, compared to pooled naïve mice of either gender (*n* = 5/sex). **C** Immune cell subtypes comprising CD45^+^CD4 + cells in hearts obtained from wild-type (WT) mice female and male mice after 18 h of polymicrobial sepsis. *p* values refer to post hoc testing after ANOVA testing for sex *x* condition interaction. Additional immune cell data are shown in Additional file 1

CD45^+^ cells infiltrated the heart after 18 h of polymicrobial sepsis (Fig. 5A) compared to naïve mice to a similar extent in female and male wild-type mice (*n* = 5/sex; *p* = 0.20; Fig. 5B). Male mice had around twice as many CD45^+^CD4^+^ leucocytes present in the heart (*p* = 0.03; Fig. 5C; Additional file 1: Supplementary data 6).

### Sex-specific cardiac gene transcription for cardioprotective and contractility pathways

**Fig. 6:**
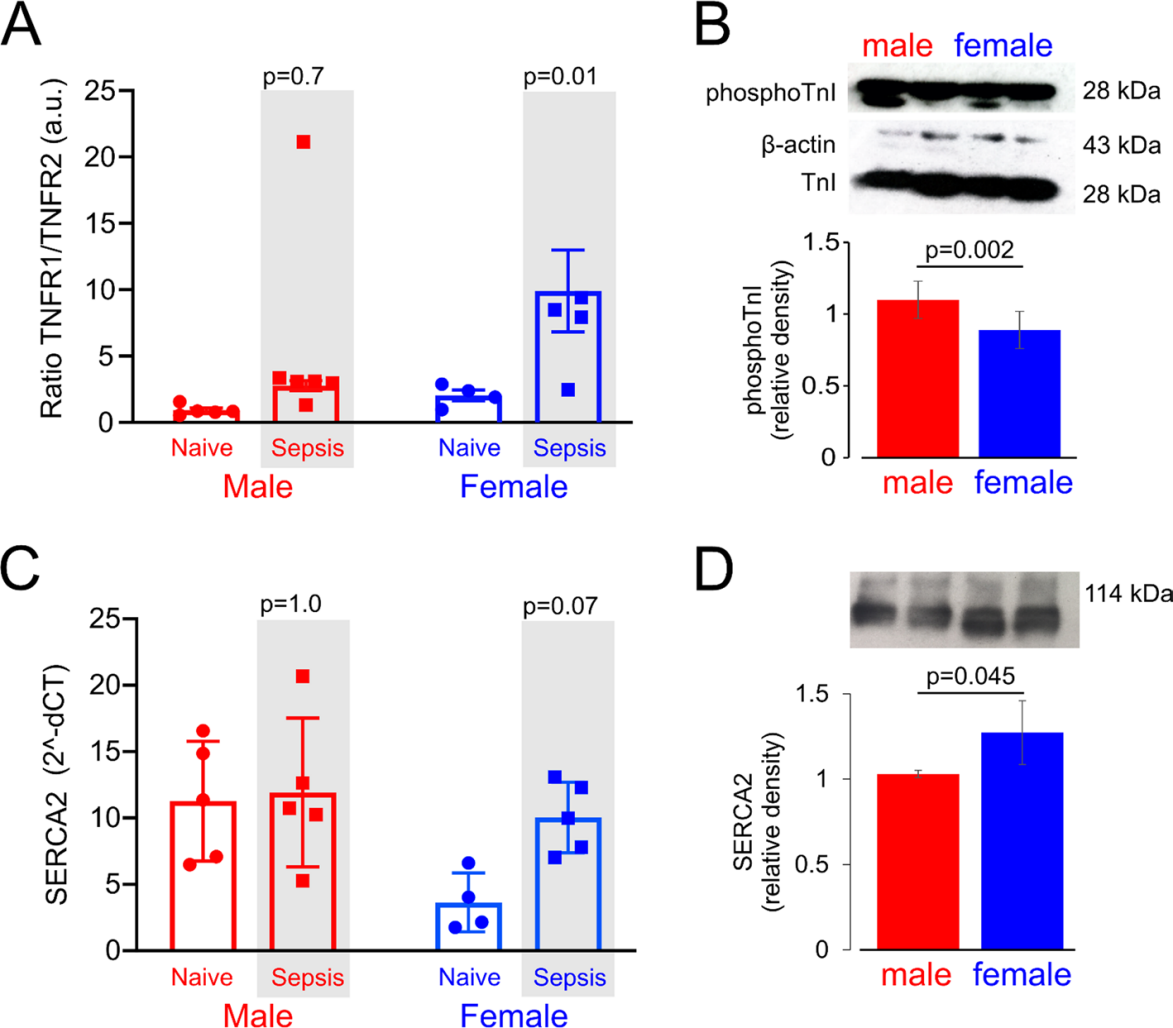
Expression of genes regulating cardioprotection and contractility after polymicrobial sepsis. **A** TNFR1/R2 ratio of mRNA expression in female and male wild-type mice before and 18 h after onset of sepsis (*n* = 6/sex). **B** Immunoblot for phosphotroponin I protein expression in left ventricle 18 h after sepsis in wild-type male and female mice (uncropped version in Additional file 1). Β-actin corrected values are compared in summary graph for 8/mice each sex. Individual *p* values on each panel refer to post hoc testing after ANOVA testing for sex *x* gene interaction. **C** mRNA expression of sarco(endo)plasmic reticulum Ca2 + ATPase 2 (SERCA2) female and male wild-type mice before and 18 h after onset of sepsis (*n* = 6/sex). Individual *p* values on each panel refer to post hoc testing after ANOVA testing for sex *x* condition interaction. **D** Immunoblot for SERCA2 protein expression in left ventricle 18 h after sepsis in wild-type male and female mice (original uncropped version in Additional file 1). Β-actin corrected values are compared in summary graph for 8/mice each sex. Individual *p* values on each panel refer to post hoc testing after ANOVA testing for sex *x* gene interaction

To further examine the cardiac molecular differences between female and male wild-type mice, we found that female wild-type mice had ~threefold increased TNFR1:R2 ratio (9.9 ± 6.9 a.u.) in left ventricular tissue, compared to male mice (2.7 ± 0.8 a.u.), which was associated with upregulated expression levels of TNFα receptor 1 mRNA (*p* = 0.01; Fig. 6A). To assess cardiac contractility at the molecular level, we estimated phosphorylation of Ser23 and Ser24 in TnI, which occurs through PKA and PKC in response to β-adrenergic stimulation (Fig. 6B)*.* Wild-type male mice had higher protein levels of phosphoTnI, compared with wild-type females [mean difference in relative density: 0.42 (95% CI: 0.19–0.66); *p* = 0.002]. SERCA2 mRNA increased in female wild-type mice 18 h after sepsis (Fig. 6C), which was associated with modestly higher protein levels compared to males (mean difference in relative density: 0.24 (95% CI: 0.01–0.47); *p* = 0.046; Fig. 6D)*.*

### Sex-specific extra-cardiac TNFα release after polymicrobial sepsis

**Fig. 7:**
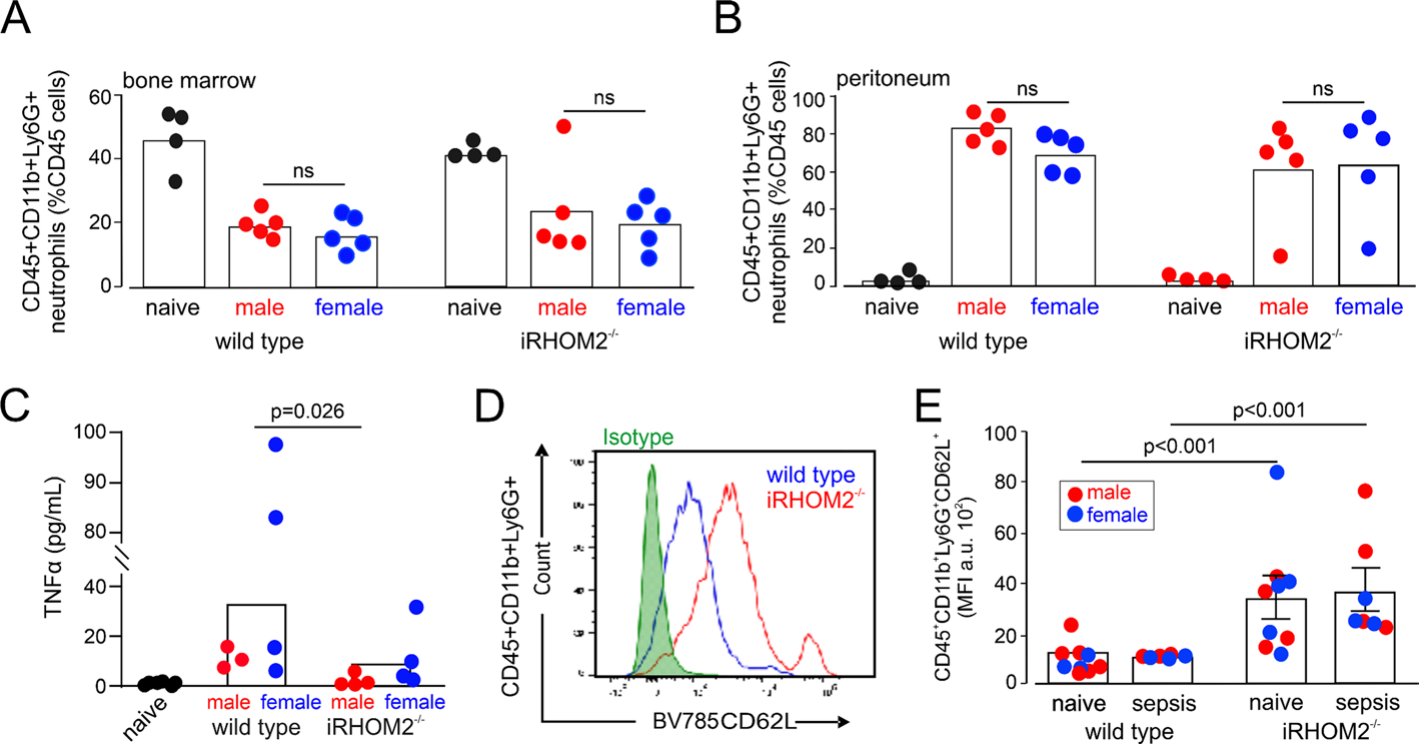
Inflammatory response in iRHOM2-deficient female and male mice after polymicrobial sepsis. **A** Bone marrow and peritoneal enumeration of neutrophils before and after polymicrobial sepsis (*n* = 4–5 mice/sex, as indicated). Similar numbers of neutrophils egressed from bone marrow and accumulated in the peritoneum 18 h after onset of sepsis. **B** Intraperitoneal TNFα protein levels were higher in wild-type mice (*n* = 7; *p* = 0.026), compared with iRHOM2-deficient mice (*n* = 8), despite similar neutrophil infiltrates. **C** Surface CD62L expression in neutrophils obtained from wild-type and iRHOM2-deficient mice. This representative data show that CD62L surface expression declines in wild-type mice after sepsis, but fails to be shed from the neutrophil cell surface in iRHOM2-deficient mice (Additional file 1: Supplementary data for summary data)

To examine whether the failure to shed TNFα from peripheral circulating leukocytes contributes to the sex-specific differences in cardiac inflammation in early sepsis, we examined polymicrobial sepsis in iRHOM2^−/−^ mice, for which there is no sexual dimorphism reported [18, 30]. Similar weight loss and glucose levels were measured in iRHOM2^−/−^ mice, compared to wild-type littermates (Table 1). Neutrophils are the key innate cell type mobilised in cecal slurry at the timepoint we examined [31]. We found that similar numbers of neutrophils migrated from the bone marrow to septic peritoneum in wild-type and iRHOM2^−/−^ mice (Fig. 7A). Higher TNFα protein levels (mean difference: 27 pg mL^−1^ (95% CI: 2–56) in peritoneal fluid after 18 h of polymicrobial sepsis were measured in wild-type, compared to iRHOM2^−/−^ mice (*p* = 0.026; Fig. 7B). Both male and female iRHOM2^−/−^ mice failed to release TNFα, with levels approaching those measured in naïve mice from both genotypes (Fig. 7B). Consistent with the inability of iRHOM2^−/−^ mice to cleave TNFα, iRHOM2^−/−^ mice of both sexes also failed to shed CD62L, a key selectin that is rapidly shed and detected at elevated levels in the plasma during sepsis and correlates with survival [32] (Fig. 7C; Additional file 1: Supplementary data 7). Neutrophil CXCR2 was higher in iRHOM2^−/−^ mice but declined to similar levels as wild-type mice after sepsis (Additional file 1: Supplementary data 7).

### Cardiovascular function in absence of shedding of TNFα by peripheral leukocytes

**Fig. 8:**
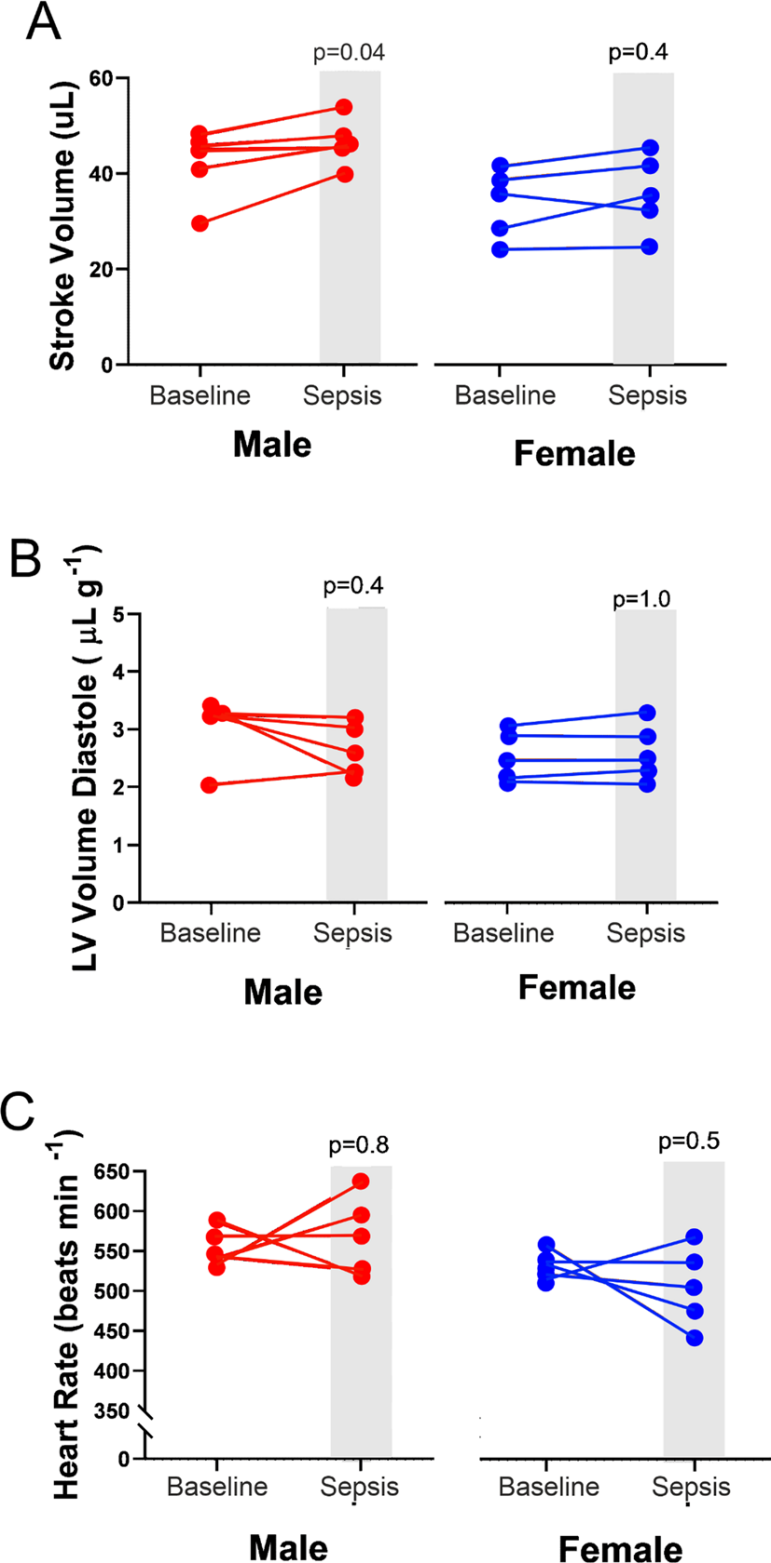
Loss of TNFα shedding in iRHOM2 deficiency and cardiac function after polymicrobial sepsis. **A** Summary data for stroke volume in male and female iRHOM2-deficient mice (*n* = 5/sex). Stroke volume increased only in both wild-type male mice. Data for each individual mouse were compared to its own pre-sepsis baseline reading, measured ~ 1 week before the onset of sepsis. Individual *p* values on each panel refer to post hoc testing after ANOVA testing for sex *x* time interaction. **B** Left ventricular volume (standardised by body weight) in iRHOM2-deficient male and female mice (*n* = 5/sex), as indicator of preload after 18 h sepsis. Data for each individual mouse were compared to its own pre-sepsis baseline reading, measured ~ 1 week before the onset of sepsis. Individual *p* values on each panel refer to post hoc testing after ANOVA testing for sex *x* time interaction. **C** Heart rate in male and female iRHOM2-deficient mice (*n* = 5/sex), as indicator of preload after 18 h sepsis. Data for each individual mouse were compared to its own pre-sepsis baseline reading, measured ~ 1 week before the onset of sepsis. Individual *p* values on each panel refer to post hoc testing after ANOVA testing for sex *x* time interaction

As in wild types, only male iRHOM2^−/−^ mice increased stroke volume after polymicrobial sepsis from 42 ± 7 µL at baseline to 47 ± 5 µL (*p* = 0.038; Fig. 8A). This difference in stroke volume between female and male iRHOM2^−/−^ mice occurred with similar changes in left ventricular end-diastolic volume (Fig. 8B) and heart rate (Fig. 8C; Additional file 1: supplementary data 8).

### Cardiac contractility and inflammation in absence of shedding of TNFα by peripheral leukocytes

**Fig. 9:**
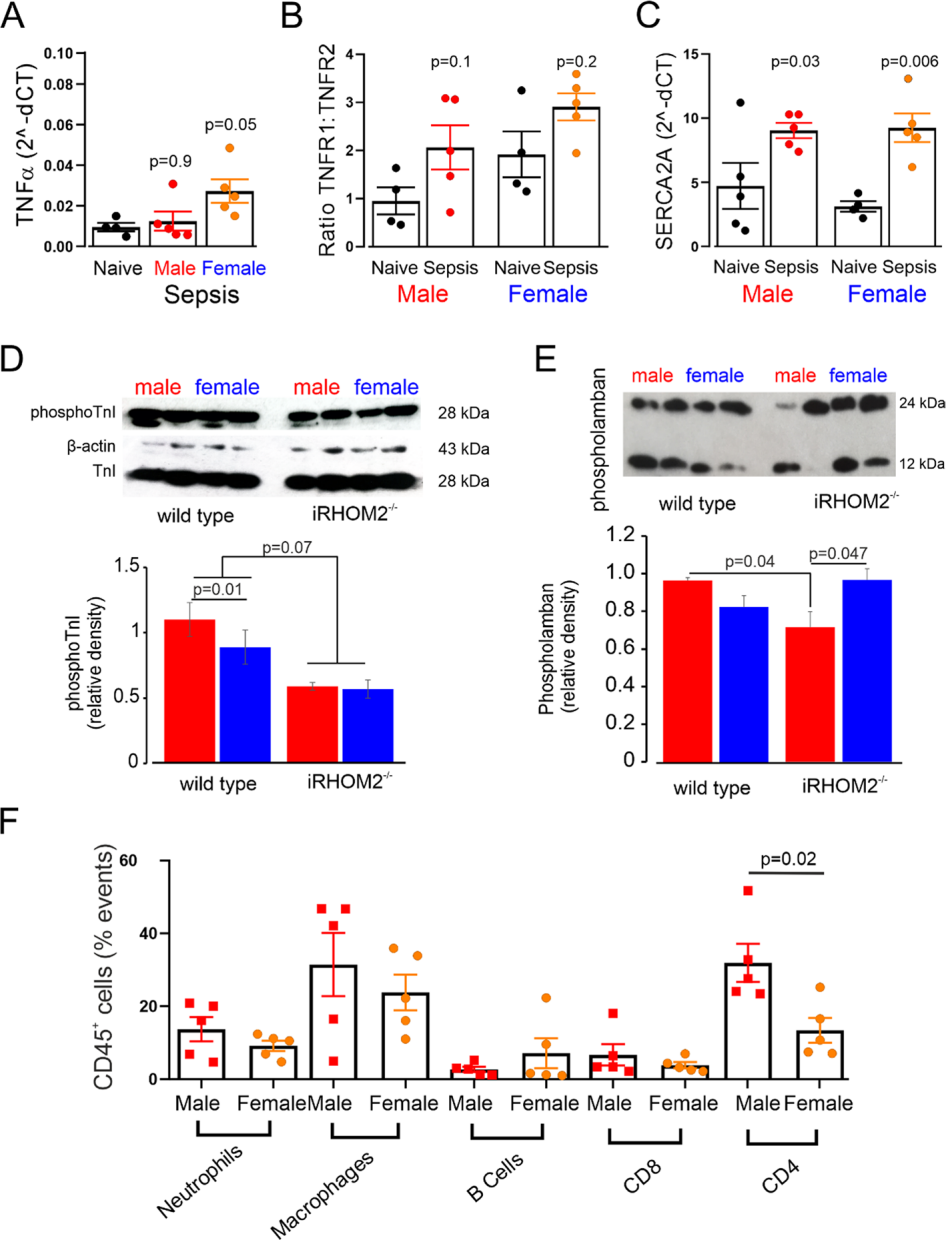
Loss of TNFα shedding in iRHOM2 deficiency and cardiac inflammation and contractility after polymicrobial sepsis. **A** Cardiac TNFα gene transcription in naïve and septic RHOM2 deficient mice (*n* = 5/sex). **B** TNFR1/R2 ratio of mRNA expression in female and male iRHOM2-deficient mice before and after sepsis (*n* = 5/sex). C mRNA expression of sarco(endo)plasmic reticulum Ca2 + ATPase 2 (SERCA2) female and male iRHOM2-deficient mice before and after sepsis (*n* = 5/sex). **D** Immunoblots showing comparison between wild-type and knockout mice for phosphotroponin I protein expression 18 h after. Β-actin corrected values are compared in summary graph for 8/mice each sex. Individual *p* values on each panel refer to post hoc testing after ANOVA testing for sex *x* gene interaction. **E** Immunoblot for phospholamban protein expression in left ventricle 18 h after sepsis in wild-type male and female mice (original uncropped version in Additional file 1). Β-actin corrected values are compared in summary graph for 8/mice each sex. Individual *p* values on each panel refer to post hoc testing after ANOVA testing for sex *x* gene interaction. **F** A higher proportion of CD4 + cells infiltrating the left ventricle in male iRHOM2^−/−^ mice (31.9 ± 5.1% CD45 + cells; *n* = 5), compared to 13.4 ± 3.4% (*n* = 5) in female iRHOM2^−/−^ mice (*p* = 0.02). Individual *p* values on each panel refer to post hoc testing after ANOVA testing for sex *x* time interaction

Female and male iRHOM2^−/−^ mice (*n* = 5/sex) had similar left ventricular expression of TNFα (male: 0.01 ± 0.01; female: 0.03 ± 0.01 a.u.; *p* = 0.80; Fig. 9A) and cardiac TNFR1: R2 ratio (male: 2.1 ± 1.0; female: 2.9 ± 0.6 a.u.; *p* = 0.20; Fig. 9B). In the absence of TNFα shedding, left ventricular SERCA2 mRNA levels increased in both sexes [male: 4.8 ± 4.0 to 9.1 ± 1.4 (*p* = 0.03); female: 3.2 ± 0.8 to 9.4 ± 2.5 a.u. (*p* = 0.006); Fig. 9C]. Similar changes were observed in SERCA2 protein levels [mean difference in relative density: 0.24 (95% CI: 0.1–0.48); *p* = 0.047; Additional file 1: Supplementary data 9]. In the absence of TNFα shedding, left ventricular phosphoTnI was similar between iRHOM2^−/−^ male and female mice (Fig. 9D). iRHOM2^−/−^ female mice had similar phosphoTnI protein expression to wild-type females, but lower levels compared to wild-type males (*p* = 0.006; Fig. 9D). iRHOM2^−/−^ males had lower levels of phospholamban protein expression than female iRHOM2^−/−^ mice and wild-type males (Fig. 9E). Left ventricular NOX-2 mRNA levels increased in iRHOM2^−/−^ females only (Additional file 1: Supplementary data 10).

These physiologic changes correlated with similar serum troponin I levels (Table 1) and immune cell infiltration in female and male iRHOM2^−/−^ hearts (*p* = 0.17 for comparison between male versus females; Additional file 1: Supplementary data 11) 18 h after the onset of sepsis. A higher proportion of CD4 + cells infiltrated the left ventricle in male iRHOM2^−/−^ mice 18 h after sepsis (31.9 ± 5.1% CD45 + cells), compared to 13.4 ± 3.4% in female iRHOM2^−/−^ mice (*p* = 0.02; Fig. 9F).

## Discussion

This series of studies adds further support to the hypothesis that females are protected from cardiac injury after systemic inflammation/sepsis. Our translational murine experimental data show that cardiac function remains at baseline levels in females, in contrast to the relative hyperdynamic response observed in males. We found that that female mice generated less cardiac TNFα-mediated inflammation and injury after a septic insult, which was associated with predominant cardioprotective TNF receptor profile. The absence of TNFα shedding from peripheral circulating leukocytes failed to prevent the cardiac physiologic differences between female and male mice. Given similar degrees of modest myocardial injury (as reflected by serum troponin I measurements), these data suggest that intrinsic mechanisms within the heart protect females during systemic inflammation. Taken together, these data suggest that understanding sex-specific mechanisms in greater detail could provide therapeutic opportunities for cardioprotection in sepsis.

Our experimental data are the first to examine early cardiac immune cell infiltrates in polymicrobial sepsis. The observation that more CD4 + leukocytes infiltrated the hearts of male mice in sepsis is consistent with a direct role for T lymphocytes in mediating acute cardiac inflammation, including autoimmune myocarditis. Dendritic cells present ingested external antigens through the major histocompatibility complex class II complex to induce CD4 + T-cell responses [33]. Adoptive transfer of purified T lymphocytes from mice with active myocarditis, ischaemic or pressure-overload heart failure results in the same cardiac pathology occurring in recipient mice [34–37]. Moreover, specific ablation of CD4 T cells reduces the initial healing and remodelling response after myocardial infarction and aortic constriction [35] through the formation of mature collagen matrix and fibrosis. Mice with transgenic CD4 + T-cell receptor specific for ovalbumin failed to develop HF and adverse remodelling [38]. Therefore, CD4 + T cells are critical for the development of cardiac dysfunction that may result in heart failure.

By attenuating the release of leucocyte TNFα using a genetic murine model, we also show that leucocyte-derived TNFα is unlikely to be a major contributor to sex-specific differences in cardiac function during the critical phase of early sepsis. In particular, male mice show a similar cardiac phenotype after sepsis in the presence, or absence, of leucocyte TNFα shedding, which suggests that intra-cardiac inflammation is key. By using a carefully calibrated experimental model with morbidity, but free from mortality, we were able to dissect the relative contributions of cardiac versus systemic inflammation. Our data suggest that non-fatal sepsis is a more potent stressor for males, as reflected by local (cardiac) inflammation, oxidative stress and increased phosphorylation of Ser23 and Ser24 in TnI, which occurs through PKA and PKC in response to β-adrenergic stimulation. The absence of TNF shedding reduced phosphotroponin protein expression, suggesting inflammation is a key driver of increased contractility. Moreover, in male wild-type mice, phospholamban protein expression was higher than in iRHOM deficient males, which may reflect its role as a prominent mediator of the β-adrenergic responses in the mammalian heart [25].

Minor changes in cardiac sarcoplasmic reticular Ca(2+)-ATPase enzyme SERCA2 suggest that additional mechanisms operate during acute stressors like sepsis contribute, which requires additional studies of calcium signalling studies in isolated cardiomyocytes [39]. Our finding that NOX-2 transcription levels were increased only in females is consistent with cardiomyocyte-specific Nox2 overexpression enhancing contractile function -at least acutely during systemic stress- through an increase in sarcoplasmic reticulum calcium uptake [24]. However, more detailed timecourse studies are also likely to reveal further insights [25]. In the clinical context, males with more extensive coronary artery disease (which may be occult) would be predicted to sustain excess myocardial injury through a higher cardiac workload and hence possible demand-ischaemia.

Several clinical trial attempts have been made to modulate systemic inflammation to limit cardiac injury. Central to this hypothesis is the assumption that circulating leucocytes are the key target. None have considered sex differences in either immunomodulation of circulating leucocytes or local, organ-specific inflammatory mechanisms. For example, high dose methylprednisolone, which produces leucocyte immunosuppression, increased cardiac injury as reflected by CK-MB measurements [40]. Of further note, although Toll-like receptor 4 is essential to preserve cardiac function and long-term survival in low-grade polymicrobial sepsis [41], Toll-like receptor signalling is similar in male and female mice after injury [42].

In human sepsis studies of cardiac dysfunction, there is likely to be selection bias since women appear less likely than men to be admitted to an ICU despite similar severity of illness [1]. Laboratory data show that severe experimental sepsis negates any sex-specific differences in cardiac physiology [43], which is likely explained by the majority of murine studies demonstrating a decline in cardiac function attributable to profound hypovolaemia [44]. This is particularly marked in the absence of fluid resuscitation, which confounds the interpretation of cardiac physiology in vivo [21]. Moreover, the majority of models have utilised lipopolysaccharide, which is a poor mimic of infectious sepsis [21]. Our murine model was calibrated to ensure systemic inflammation and lack of hypovolaemia without mortality. Hyperdynamic physiology appears to be an independent predictor of mortality independent of preload as a likely contributory factor [5]. Although impaired left ventricular relaxation may also reduce preload, diastolic dysfunction appears to be common when myocardial contractility is depressed in sepsis [5]. By using a model where surgery (such as caecal ligation and puncture) requiring anaesthesia could be avoided, further confounding factors that may have blurred sex-specific differences in previous studies were minimised.

Adopting a multi-platform investigation (PCR, flow cytometry) coupled with serial high-resolution ultrasound imaging enabled repeated and concomitant measures in the same mouse, thereby minimising the number of mice required to demonstrate physiologically relevant differences. Although our murine study did not consider older age or the oestrus cycle of female mice, survival in females after caecal ligation and puncture does not appear to correlate with a specific stage of the oestrus phase [43]. Murine vaginal cytology does not accurately reflect circulating estrogens [45]. Therefore, controlling for estrus cycle phases would requires a more invasive approach which would have interfered with our experimental design that aimed to minimise stressful interventions (e.g., intraperitoneal slurry rather than surgery for cecal ligation and puncture). Blood pressure monitoring (e.g., using telemetry probes) may have added further information, although given the lack of change in left ventricular volume and heart rate it seems unlikely that arterial pressure was a significant contributor to the cardiac phenotype observed. All echocardiography was performed by the same operator utilising a standard protocol for image acquisition and quality control, which minimised interobserver variability. Further, an additional analyser masked to experimental details independently verified the results. However, we cannot rule out that other echocardiographic measures (e.g., strain echocardiography) may provide additional insights [46].

We also cannot rule out a role for cardiac-resident macrophages which may differ in iRHOM2 phenotype. In male mice higher CD4^+^ cell counts were observed in cardiac tissue, suggesting that recruitment of reparative CD4^+^ subtypes [e.g., splenic CD4 + AT2R + (angiotensin-2 receptor) regulatory T cells] may be recruited to limit myocardial injury [47]. We acknowledge that genome-wide transcriptomic analyses are likely to reveal further detailed mechanistic insights into sex-specific cardiac changes in early sepsis, including a possible role for gamma-delta T cells [48].

## Conclusions

Male mice demonstrate a relative hyperdynamic cardiac function in normovolaemic sepsis, compared to females. Reduced cardiac inflammation in females, in combination with an upregulation of cardioprotective TNF receptor expression, underpins this sex-specific difference. Systemic inflammation alone does not determine this male hyperdynamic phenotype, since similar hyperdynamic changes in cardiac function occur independently of TNFα shedding from circulating leukocytes. Taken together, our data reveal that sex-specific studies help reveal mechanistic insights that may provide new therapeutic avenues to minimise organ dysfunction and accelerate recovery from sepsis.

## Supplementary Information

**Additional file 1.** Expanded methods (including ARRIVE statement) and additional results.

## Data Availability

On request from GLA—g.ackland@qmul.ac.uk.

## Abbreviations

TNFα: Tumour necrosis factor
TNFR1: TNF receptor 1
TNFR2: TNF receptor 2
ADAM17: ADAM Metallopeptidase Domain 17
CD62L: L-selectin
iRHOM2: Inactive rhomboid protein 2
NOX-2: NADPH oxidase 2
SERCA2: Sarco/endoplasmic reticulum Ca(2+)-ATPase
NF-ĸB: Nuclear factor-ĸB
SOD-1: Superoxide dismutase
CD45: Protein tyrosine phosphatase receptor type C
mRNA: Messenger RNA
