# Supplementary material for: Sex-specific differences in cardiac function, inflammation and injury during early polymicrobial sepsis
Source: Intensive Care Med Exp. 2022 Jun 20;10:27. doi: 10.1186/s40635-022-00454-7 (PMC9209626; doi:10.1186/s40635-022-00454-7)

**Sex-specific differences in cardiac inflammation and function after sepsis.**

SUPPLEMENTARY DATA.

Sophie LM Walker, Chand Muthoo, Jenifer Sanchez, Ana Gutierrez Del Arroyo, Gareth L. Ackland

Contents

[Supplementary data 1: ARRIVE guideline adherence [including numbers of mice used and body weight]. 2](#_Toc104205725)

[Supplementary Data 2: clinical assessment and severity in murine sepsis model. 4](#_Toc104205726)

[Supplementary Data 3: flow cytometry antibodies. 4](#_Toc104205727)

[Supplementary Data 4: flow cytometry gating strategy. 5](#_Toc104205728)

[Supplementary data 5. PCR primers. 6](#_Toc104205729)

[Supplementary Data 6: Cardiac flow cytometry in wild-type mice. 6](#_Toc104205730)

[Supplementary Data 7: Neutrophil CXCR2 in wildtype and iRHOM2 KO mice. 7](#_Toc104205731)

[Supplementary Data 8: cardiac echo data in wild-type and iRHOM2 KO mice. 7](#_Toc104205732)

[Supplementary Data 9: uncropped immunoblots. 8](#_Toc104205733)

[Supplementary Data 10: NOX-2 mRNA in naïve and septic wild-type and iRHOM2 knockout mice. 8](#_Toc104205734)

[Supplementary Data 11: confirmation of gene knockout and cardiac immune cell infiltrates in iRHOM2 knockout mice. 9](#_Toc104205735)

# Supplementary data 1: ARRIVE guideline adherence [including numbers of mice used and body weight].

|  | | ITEM | RECOMMENDATION | Section/ Paragraph | |
| --- | --- | --- | --- | --- | --- |
| 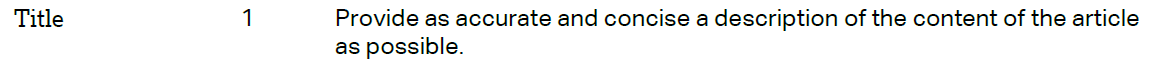 | | | Page 1 | |
| 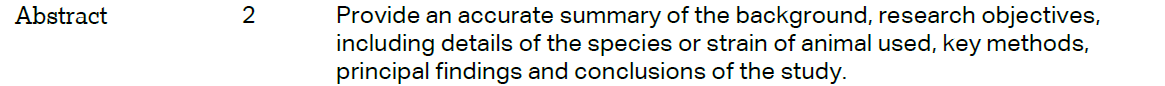 | | | Page 4 | |
| INTRODUCTION | | |  | |
| 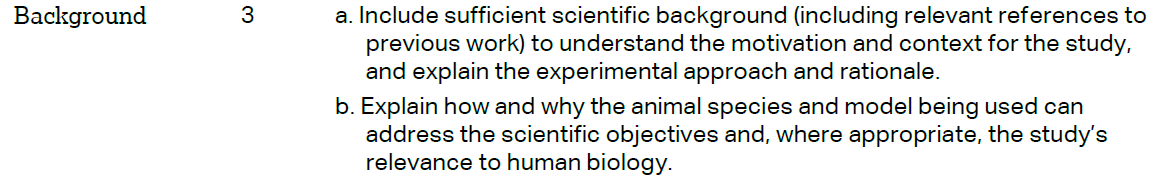 | | | Page 6 | |
| 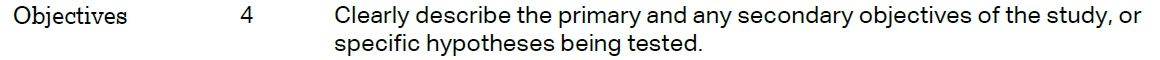 | | | Page 46-7 | |
| METHODS | | |  | |
| 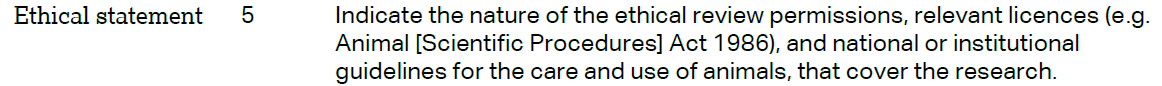 | | | Page 9 | |
| 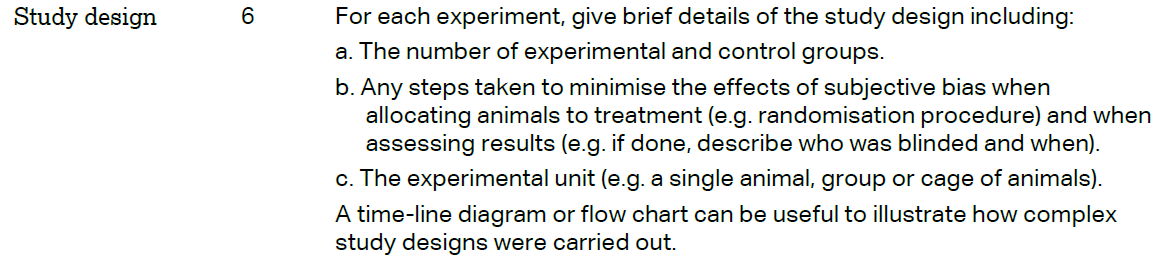 | | | Page 9-11 | |
| 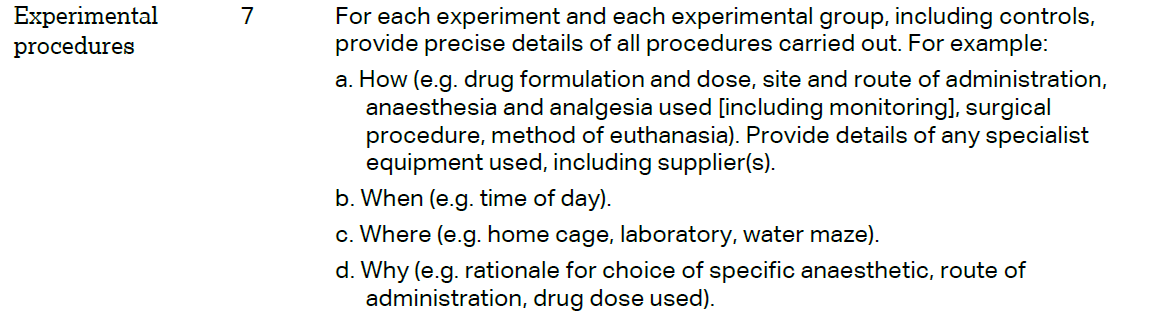 | | | Page 9-11  methods | |
| 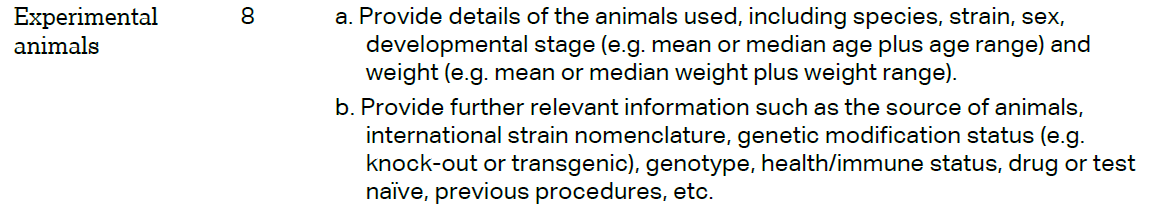 | | | Page 9-11 | |
| 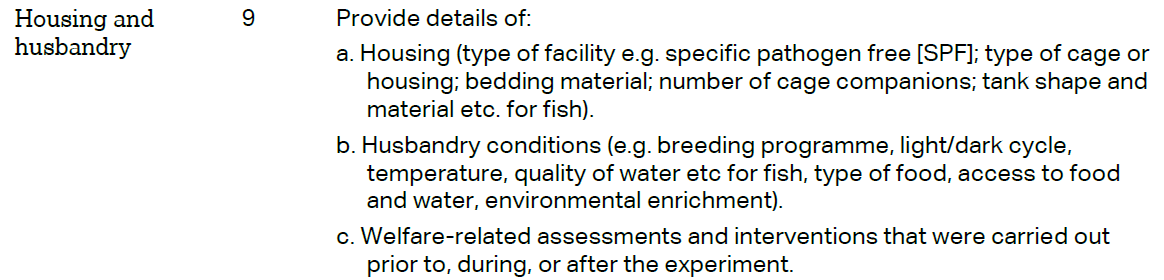 | | | Page 9 | | |
| 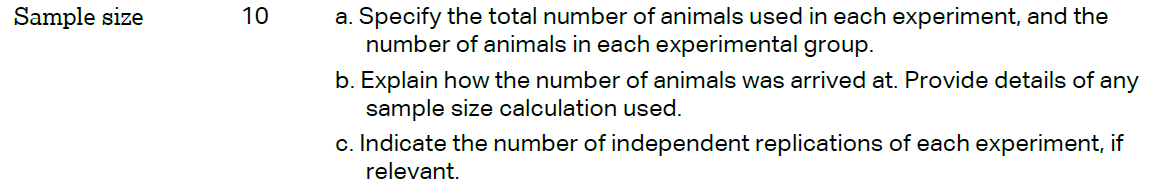 | | | Page 11 | | |
| 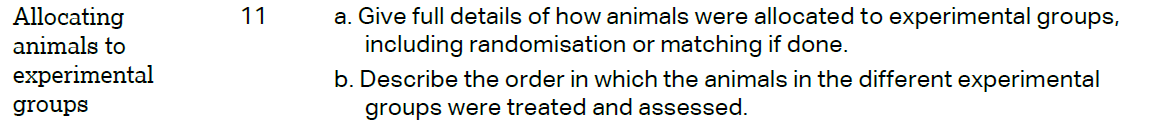 | | | Page 11 | | |
| 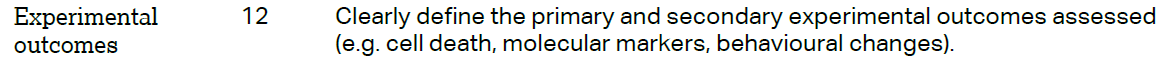 | | | Page 10 | | |
| 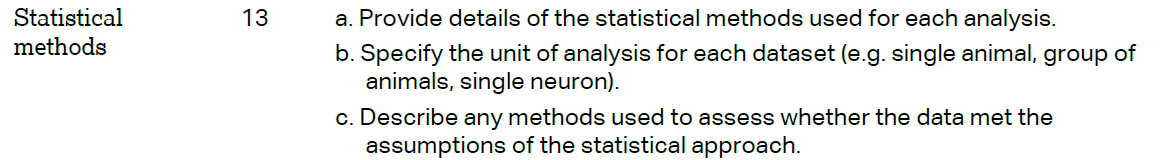 | | | Page 11 | | |
| RESULTS | | |  | | |
| 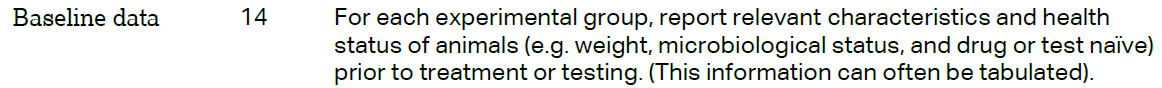 | | | Page 13 | | |
| 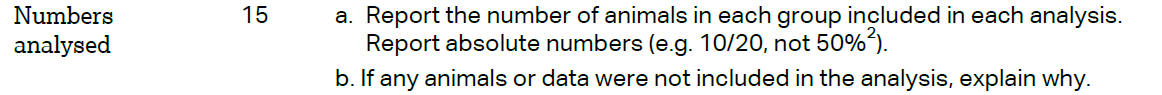 | | | Page 13 | | |
| 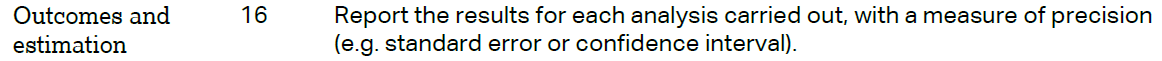 | | | Page 13-14 | | |
| 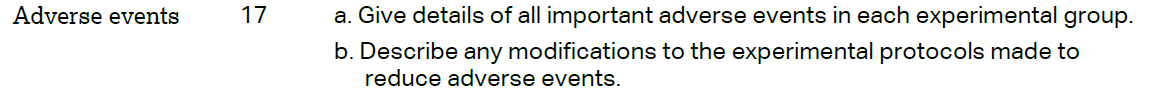 | | | Page 13, suppl data. | | |
| DISCUSSION | | |  | | |
| 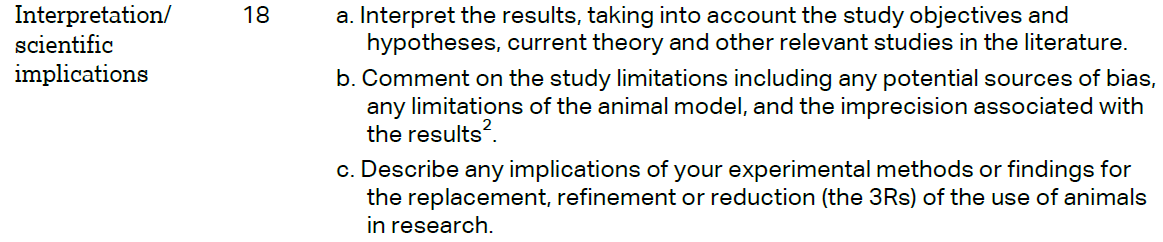 | | | Page 15-16 | | |
| 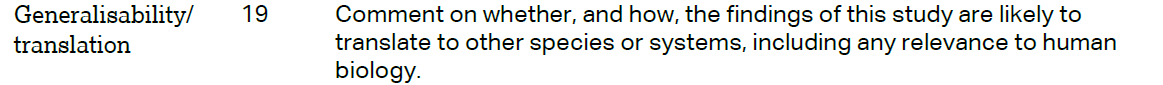 | | | Page 16-17 | | |
| 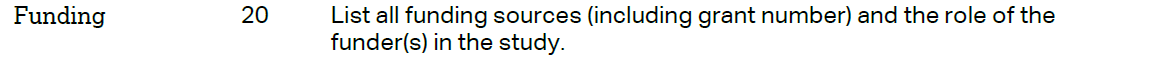 | | | | Page 2 |

**Numbers of mice used (including baseline body weight)**

Numbers used for echo/ PCR/ blots and flow cytometry.

All data in grams; p values refer to genotype comparisons of weight for each sex

**Male**: WT: 28.1±0.9, n=16. KO: 28.7±0.8, n=15. [p=0.65]

**Female**: WT: 23.4.5±0.9, n=17. KO: 22.4±0.6, n=15. [p=0.3].

# Supplementary Data 2: clinical assessment and severity in murine sepsis model.

| **Severity Scoring System** | | |  |  |  |
| --- | --- | --- | --- | --- | --- |
| **Mild** | | **Moderate** | | **Severe** | |
| Appearance | Alertness | Appearance | Alertness | Appearance | Alertness |
| Hunched  Piloerect  No Bloating | Alert  Occasional interest in environment  Moves freely | Hunched  Marked piloerection  Bloated abdomen  Sunken eyes | Depressed level of alertness  Little interest in environment  Moves with difficulty | Marked piloerection  Marked bloated abdomen  Conjunctival injection | Marked depressed (or absent) level of alertness  No interest in environment  No movement |

# Supplementary Data 3: flow cytometry antibodies.

| Antigen | Fluorochrome | Excitation laser (nm) | Isotype | Manufacturer |
| --- | --- | --- | --- | --- |
| CD45 | FITC | B530/30 | Rat IgG2b | Miltenyi |
| CD8a | PerCPVio700 | B695/40 | Rat IgG2a | Miltenyi |
| CD4 | APC | R670/14 | Rat IgG2a | Miltenyi |
| Ly6G | APCVio770 | R780/60 | REA | Miltenyi |
| Ly6C | VioBlue | V450/50 | Rat IgG2a | Miltenyi |
| CD45B220 | VioGreen | V525/50 | Rat IgG2a | Miltenyi |
| CD62L | Brilliant Violet 785 | V780/60 | Rat IgG2a | Biolegend |
| F4/80 | PE | YG582/15 | Rat IgG2a | Miltenyi |
| CD11b | PEVio615 | YG610/20 | REA | Miltenyi |
| CXCR2 | PEVio770 | YG780/60 | Rat IgG2b | Miltenyi |

# Supplementary Data 4: flow cytometry gating strategy.


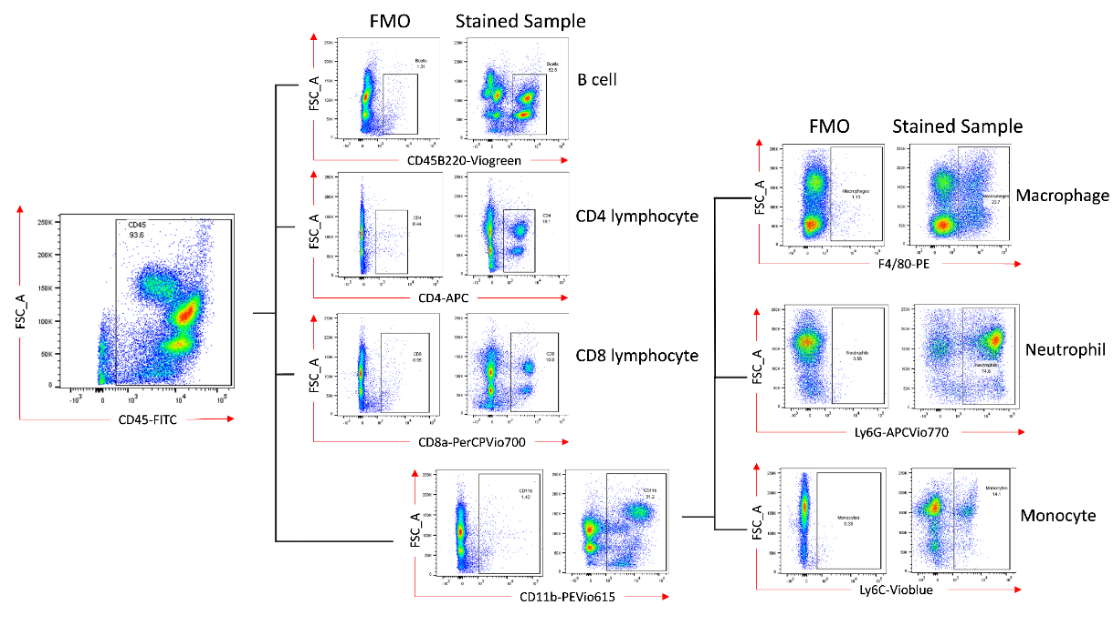


# Supplementary data 5. PCR primers.

Primers provided by IDT Technology , Leuven, Belgium

| Gene | Forward Sequence (5’-3’) | Reverse Sequence (3’-5’) |
| --- | --- | --- |
| ANP | TGATAGATGAAGGCAGGAAGCCGC | AGGATTGGAGCCCAGAGTGGACTAGG |
| SOD1 | AACCAGTTGTGTTGTCAGGAC | CCACCATGTTTCTTAGAGTGAGG |
| NFkB | ATGGCAGACGATGATCCCTAC | TGTTGACAGTGGTATTTCTGGTG |
| TNFα | GAACTGGCAGAAGAGGCACT | GGTCTGGCCCATAGAACT |
| TNFR1 | CCGGGAGAAGAGGGATAGCTT | TCGGACAGTCACTCACCAAGT |
| TNFR2 | ACACCCTACAAACCGGAACC | AGCCTTCCTGTCATAGTATTCCT |
| HRPT1 | GCTTGCTGGTGAAAAGGACCTCTCGAAG | CCCTGAAGTACTCATTATAGTCAAGGGCAT |

# Supplementary Data 6: Cardiac flow cytometry in wild-type mice.


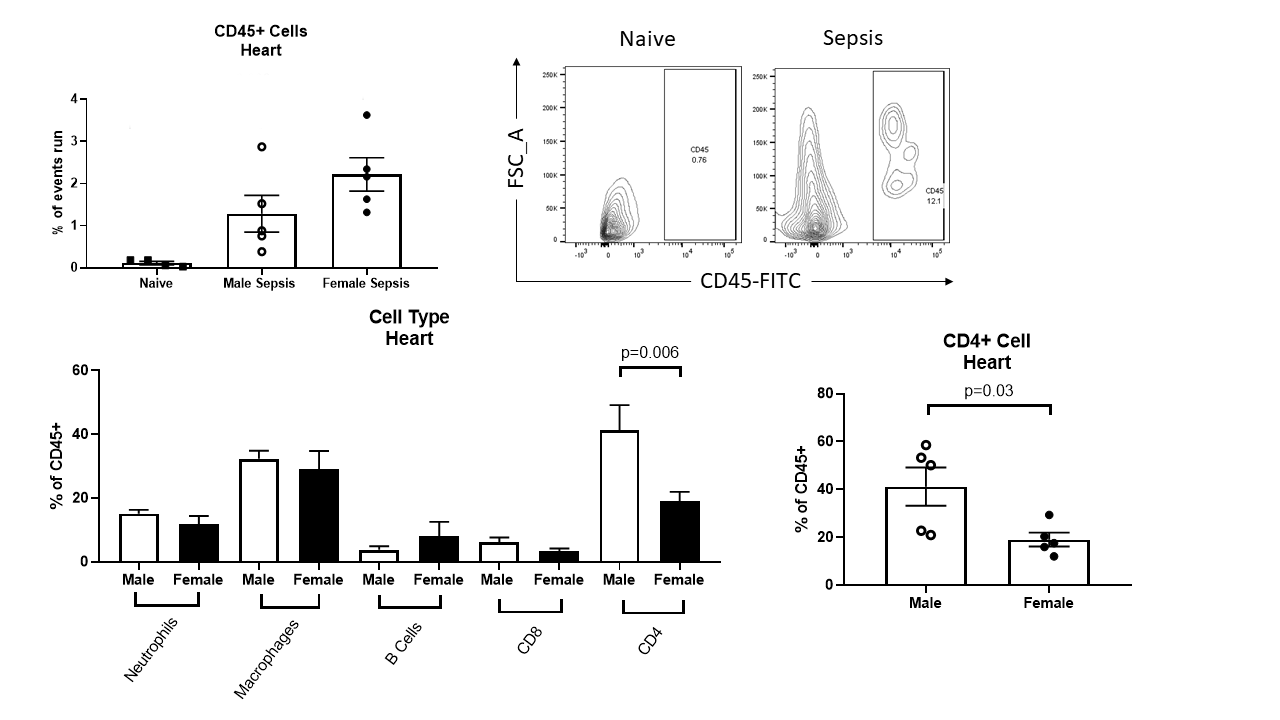


# Supplementary Data 7: Neutrophil CXCR2 in wildtype and iRHOM2 KO mice.


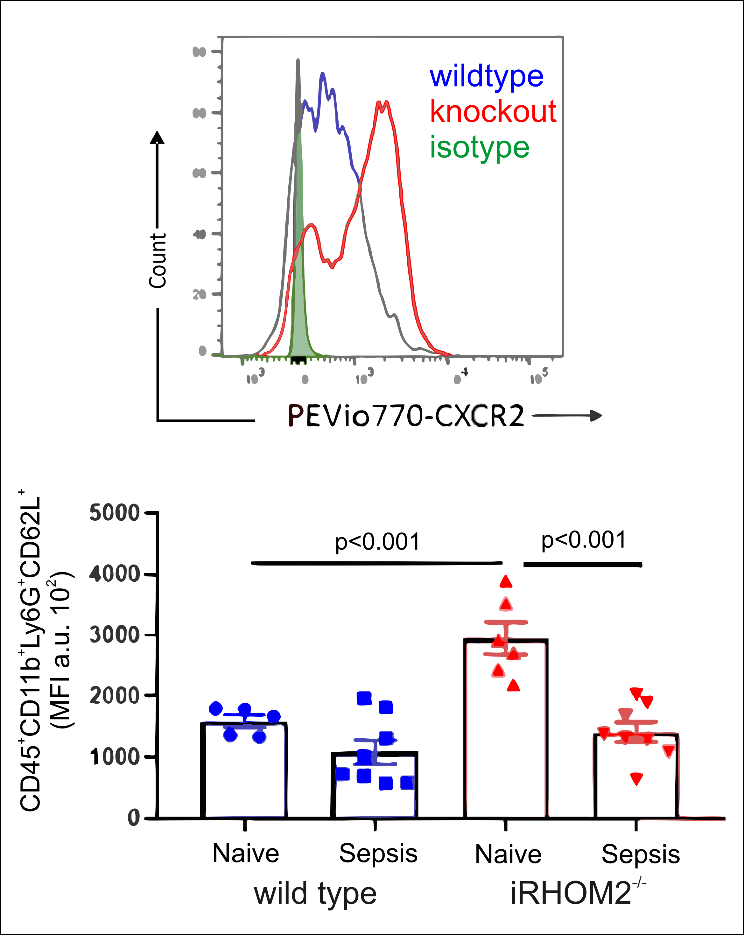


# Supplementary Data 8: cardiac echo data in wild-type and iRHOM2 KO mice.

Asterisk refers to P<0.05, by repeated measures ANOVA (baseline x sepsis for each individual mouse)

|  |  | Baseline (mean±SD) | Sepsis (mean±SD) | P value |
| --- | --- | --- | --- | --- |
|  | Wild type |  |  |  |
| Ejection Fraction (%) |  | Male: 63±5  Female: 65±7 | Male:72±5  Female: 70±7 | Male: 0.01*  Female: 0.3 |
| Fractional Shortening (%) |  | Male: 35±3  Female: 34±4 | Male: 43±8  Female:39±6 | Male: 0.03*  Female: 0.2 |
| Cardiac output (ml/min) |  | Male: 21±2  Female: 18±4 | Male: 23±3  Female: 19±5 | Male: 0.06  Female: 0.99 |
|  | KO iRHOM2 |  |  |  |
| Ejection Fraction (%) |  | Male: 57±1  Female: 68±7 | Male: 61±3  Female: 68±7 | Male: 0.5  Female: 0.99 |
| Fractional Shortening (%) |  | Male: 29±1  Female: 37±5 | Male:32±2  Female: 37±6 | Male: 0.6  Female: 0.99 |
| Cardiac output (ml/min) |  | Male: 23±4  Female: 19±3 | Male:26±3  Female: 18±4 | Male: 0.08  Female: 0.97 |

# Supplementary Data 9: uncropped immunoblots.


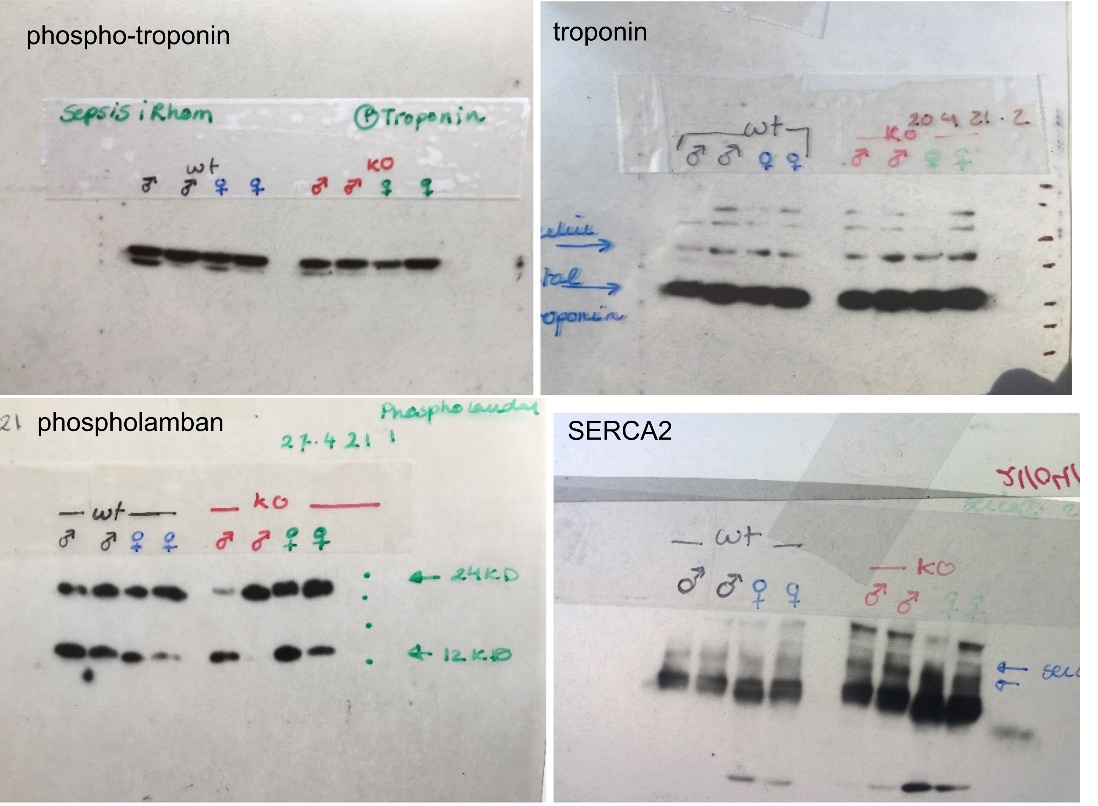
.

# Supplementary Data 10: NOX-2 mRNA in naïve and septic wild-type and iRHOM2 knockout mice.

All p values refer to naïve versus sepsis comparison, by ANOVA.


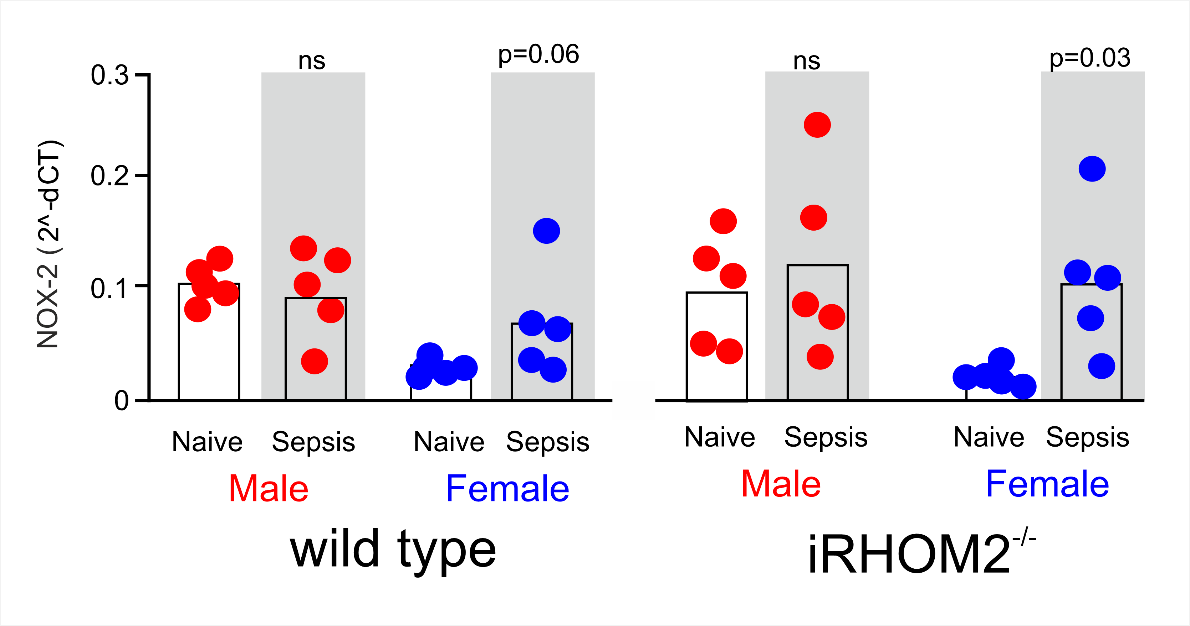


# Supplementary Data 11: confirmation of gene knockout and cardiac immune cell infiltrates in iRHOM2 knockout mice.


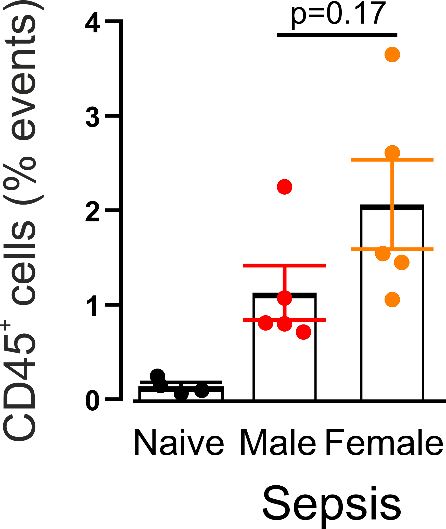

Supplement: Supplementary file 1 — Additional file 1. Expanded methods (including ARRIVE statement) and additional results. [file 40635_2022_454_MOESM1_ESM.docx]
